# Supplementary material for: Impact of BNT162b First Vaccination on the Immune Transcriptome of Elderly Patients Infected with the B.1.351 SARS-CoV-2 Variant
Source: Res Sq. 2021 Jun 1:rs.3.rs-509143. Preprint. [Version 1] doi: 10.21203/rs.3.rs-509143/v1 (PMC8183022; doi:10.21203/rs.3.rs-509143/v1)
Supplement: Supplement 4 [file d8c3602c6b535c347be59013.pdf]

**Supplementary Information**

# Impact of BNT162b first vaccination on the immune transcriptome of elderly patients infected with the B.1.351 SARS-CoV-2 variant

Ludwig Knabl[1,*,†], Hye Kyung Lee[2,*,†], Manuel Wieser[1], Anna Mur[3], August Zabernigg[3], Ludwig Knabl Sr.[4], Simon Rauch[5], Matthias Bock[5, 6], Jana Schumacher[7], Norbert Kaiser[7], Priscilla A. Furth[8,†] and Lothar Hennighausen[2,†]

[1]TyrolPath, Zams, Austria; [2]National Institute of Diabetes, Digestive and Kidney Diseases, Bethesda, MD 20892, USA; [3]Division of Internal Medicine, Krankenhaus Kufstein, Kufstein, Austria; [4]Krankenhaus St. Vinzenz, Zams, Austria; [5]Division of Anesthesia and Intensive Care Medicine, Krankenhaus Meran, Meran, Italy; [6]Department of Anesthesiology, perioperative Medicine and Intensive Care Medicine, Paracelsus Medical University, Salzburg, Austria; [7]Division of Internal Medicine, Krankenhaus St. Johann, St. Johann, Austria; [8]Departments of Oncology & Medicine, Georgetown University, Washington, DC, USA.

*equal contribution

†Corresponding authors: LK: Ludwig.knabl@tyrolpath.at; HKL: hyekyung.lee@nih.gov; PAF: paf3@georgetown.edu; LH: lotharh@nih.gov

**Supplementary Tables**

**Supplementary Table 1.** Demographic and Clinical Characteristics of study population.

**Supplementary Table 2.** List of all genes with normalized read counts in each replicate at non-COVID controls and B.1.351 variant infected COVID-19 patients, $\log_2$ (fold change), *p*-value and adjusted *p*-value as well as upregulated gene list and GSEA analysis.

**Supplementary Table 3.** List of all genes with normalized read counts in each replicate at unvaccinated and vaccinated groups, $\log_2$ (fold change), *p*-value and adjusted *p*-value as well as upregulated gene list and GSEA analysis.

**Supplementary Table 4.** List of all genes with normalized read counts in each replicate at $1^{st}$ and $2^{nd}$ samples in vaccinated group, $\log_2$ (fold change), *p*-value and adjusted *p*-value as well as upregulated gene list and GSEA analysis.

**Supplementary Table 5.** List of all genes with normalized read counts in each replicate at unvaccinated and young-unvaccinated groups, $\log_2$ (fold change), *p*-value and adjusted *p*-value as well as upregulated gene list and GSEA analysis.

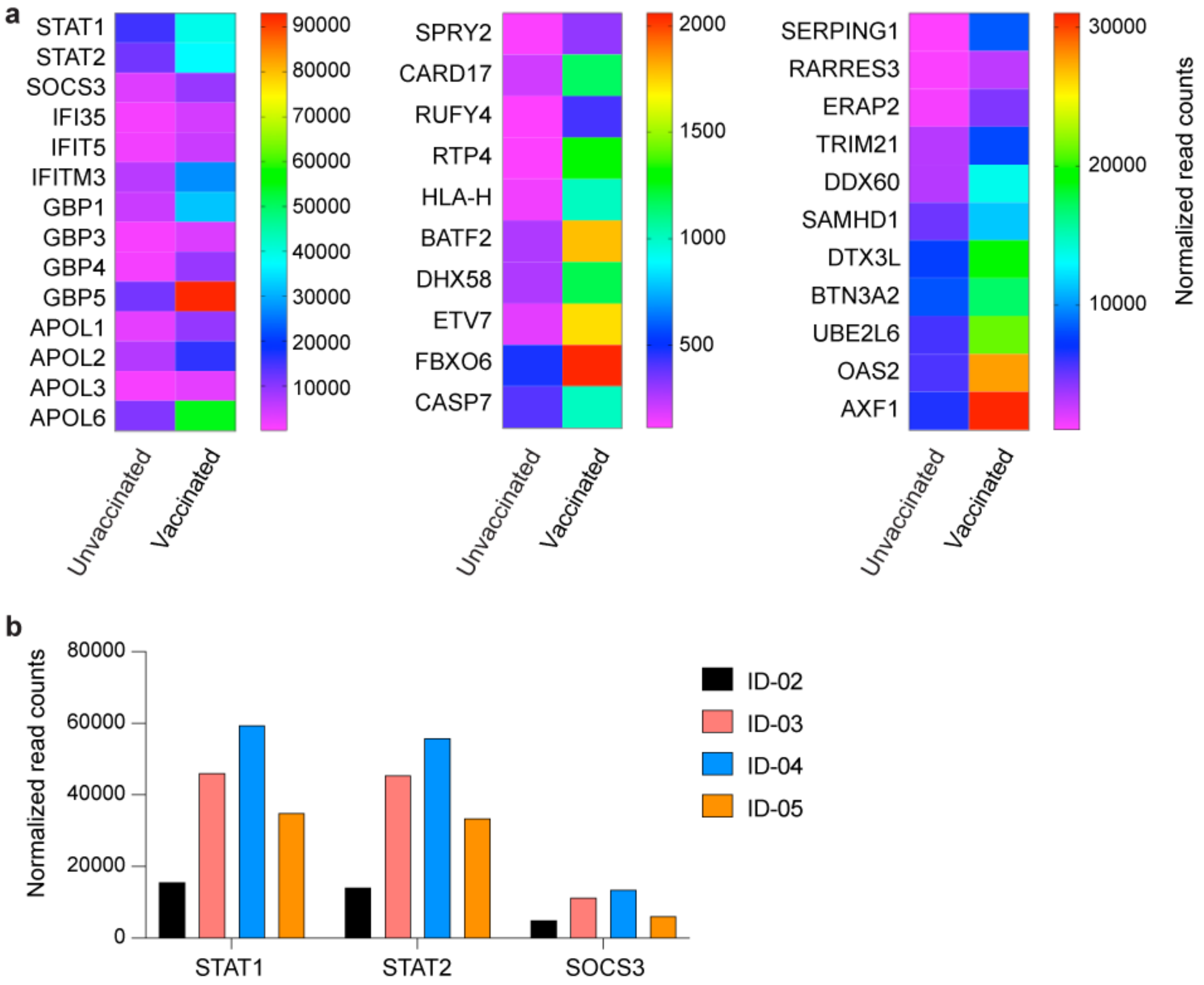


**Supplementary Figure 1. Upregulation of Interferon stimulated genes (ISGs), innate antiviral immunity and COVID-19-SARS-CoV-2 infection genes in vaccinated COVID-19 patients. a.** Heatmap showing the significant enriched genes related to immune responses and SARS-CoV-2 virus infection. **b.** mRNA levels of JAK/STAT signaling components, STAT1, STAT2 and SOCS3, measured by RNA-seq were presented by bar graphs.
